# Understanding non-psychiatric nurses’ experiences of caring for patients with mental-physical comorbidity: a descriptive phenomenological study

**DOI:** 10.3389/fpsyt.2026.1737278

**Published:** 2026-10-05

**Authors:** Jingxuan Zhang, Yaping Feng, Hong Luo, Bowen Xue

**Affiliations:** 1 Affiliated Mental Health Center & Hangzhou Seventh People’s Hospital, Zhejiang University School of Medicine, Hangzhou, Zhejiang, China; 2 School of Health Care Technology, Dalian Neusoft University of Information, Dalian, China; 3 Affiliated Hospital of Hangzhou Normal University, Hangzhou, Zhejiang, China

**Keywords:** descriptive phenomenology, mental health nursing, mental-physical comorbidity, non-psychiatric nurses, nursing care

## Abstract

**Purpose:**

This study aimed to explore non-psychiatric nurses’ experiences of caring for patients with mental-physical comorbidity, the challenges they encounter, and the factors shaping these experiences.

**Design:**

A descriptive phenomenological design was used, with data collected through face-to-face, semi-structured interviews.

**Methods:**

Between March and April 2025, 20 registered nurses from non-psychiatric departments of a tertiary hospital in Hangzhou, China, participated in in-depth interviews. Data were analysed using Colaizzi’s seven-step phenomenological method, with NVivo 12.0 used to support data management.

**Results:**

Nurses described difficulty recognising psychiatric symptoms, balancing mental and physical care, managing safety concerns, communicating with patients, and providing care during night shifts. They also reported limited intervention options and reliance on experiential knowledge. Factors shaping these experiences included stigma-related concealment of mental illness, difficulty engaging some patients in care, limited mental health knowledge and communication skills, lack of standardised guidance, weak collaboration with psychiatric services, and workload and time pressure.

**Conclusion:**

Non-psychiatric nurses described caring for patients with mental-physical comorbidity as challenging when psychiatric symptoms were difficult to recognise, mental and physical priorities competed, and access to guidance or psychiatric support was limited. Training in mental health assessment and communication, practical care protocols, and accessible psychiatric consultation may help address these difficulties.

## 1 Background

Patients with co-occurring mental and physical health conditions are encountered across healthcare systems and may require care that addresses both sets of needs. In this study, mental-physical comorbidity refers to the co-occurrence of a diagnosed mental disorder and a physical health condition in the same patient. The patients discussed by participants were admitted to non-psychiatric units primarily for treatment of a physical condition. Globally, one in eight people is affected by a mental health condition (1), and mental illness is associated with substantial personal, family, and healthcare burden (2, 3). In general hospitals, these overlapping needs create a practical nursing challenge because physical treatment is often the immediate priority.

Mental and physical illnesses are closely associated (4). People with mental disorders have higher rates of physical disease, and mortality is more than twice that of the general population (5, 6). Conversely, chronic physical illness is associated with depression and other common mental disorders (7, 8). Estimates suggest that 30–50% of patients in emergency departments have co-occurring mental and physical conditions (9, 10). About half of people with mental disorders have at least one physical comorbidity, and 35% have undiagnosed physical symptoms (11). These comorbidities are associated with greater healthcare use, longer hospital stays, and higher costs (11, 12).

Patients with mental-physical comorbidity may be admitted to general hospitals because the immediate reason for care is a physical illness, while stigma may reduce disclosure of psychiatric histories (13). Mental health needs can therefore be less visible during routine assessment. A longitudinal study of patients hospitalised with severe COVID-19 found that loneliness, social relationships, and family communication during admission were associated with depression and anxiety two years later (14). Studies also report lower treatment adherence and less engagement in self-care among some patients with co-occurring conditions (15–18), which can complicate assessment and care planning.

Research suggests that non-psychiatric nurses may prioritise physical symptoms and have difficulty recognising or responding to concurrent mental health needs (19–21). Psychiatric symptoms such as disordered thinking, unusual speech or behaviour, and emotional dysregulation can further complicate routine care (19). Patients with mental-physical comorbidity may also be perceived as “difficult”, which has been associated with less positive nursing attitudes and may affect care quality and nurse-patient interactions (22, 23).

Existing studies describe gaps in nurses’ mental health knowledge and preparedness, but provide less detail on how non-psychiatric nurses experience these overlapping demands in routine care. This study therefore used a descriptive phenomenological approach to explore nurses’ experiences of caring for patients with mental-physical comorbidity, the challenges encountered, and the factors shaping these experiences.

## 2 Methods

### 2.1 Study design

A descriptive phenomenological design was used to explore how non-psychiatric nurses experienced caring for patients with mental-physical comorbidity. This design was selected because the study sought a close description of participants’ experiences in non-psychiatric clinical settings. The study was reported in accordance with the Consolidated Criteria for Reporting Qualitative Research (COREQ) (24). Data were collected through face-to-face, semi-structured interviews.

### 2.2 Study setting

The study was conducted between March and April 2025 at a tertiary hospital in Hangzhou, China.

### 2.3 Sampling and recruitment

Purposive sampling was used to recruit non-psychiatric nurses with experience relevant to the research question. Non-psychiatric nurses were defined as registered nurses working in clinical units other than psychiatric wards. For this study, mental-physical comorbidity was operationally defined as a diagnosed mental disorder co-occurring with a physical health condition; the patients discussed had been admitted to a non-psychiatric unit primarily for treatment of the physical condition.

### 2.4 Sample size determination

Recruitment continued until additional interviews yielded no new themes relevant to the study aim (25). This point was reached after the 20th interview, and all 20 interviews were included in the analysis.

#### 2.4.1 Inclusion criteria:

(1) registered nurse; (2) at least two years of nursing experience; (3) experience independently caring for a patient with a diagnosed mental disorder who had been admitted to a non-psychiatric unit primarily for treatment of a physical condition; and (4) willingness to participate voluntarily.

#### 2.4.2 Exclusion criteria:

(1) nurses in residency training, continuing education, or internships; (2) psychiatric nurses; (3) nurses on maternity, sick, or study leave; and (4) withdrawal before completion of the interview.

### 2.5 Research instruments

#### 2.5.1 Demographic questionnaire

A brief demographic questionnaire was completed before the interviews and recorded age, gender, education level, years of nursing experience, professional title, and department.

All 20 participants were non-psychiatric nurses. Their mean age was 37.25 years (SD = 5.06), and their mean duration of nursing experience was 15.05 years (SD = 4.99). Nineteen participants were female (95%) and one was male (5%). The demographic characteristics of the participants are presented in **Table 1**.

**Table 1:** Participant characteristics (n = 20).

ID	Age	Gender	Education	Years as RN	Professional title	Unit
N1	40	Female	Bachelor’s degree or above	16	Senior nurse	Surgery
N2	29	Female	Bachelor’s degree or above	7	Registered nurse	Internal Medicine
N3	42	Female	Bachelor’s degree or above	21	Supervisor nurse	Internal Medicine
N4	43	Female	Bachelor’s degree or above	22	Supervisor nurse	Surgery
N5	45	Female	Bachelor’s degree or above	21	Supervisor nurse	Paediatrics
N6	42	Female	Bachelor’s degree or above	20	Senior nurse	Internal Medicine
N7	40	Female	Bachelor’s degree or above	20	Senior nurse	Internal Medicine
N8	35	Female	Bachelor’s degree or above	12	Registered nurse	Internal Medicine
N9	40	Female	Bachelor’s degree or above	17	Senior nurse	Surgery
N10	37	Female	Bachelor’s degree or above	14	Senior nurse	Obstetrics and Gynaecology
N11	36	Female	Bachelor’s degree or above	13	Senior nurse	Internal Medicine
N12	40	Female	Bachelor’s degree or above	17	Senior nurse	Surgery
N13	40	Female	Bachelor’s degree or above	18	Senior nurse	Obstetrics and Gynaecology
N14	31	Female	Bachelor’s degree or above	11	Senior nurse	Outpatient Service
N15	39	Male	Bachelor’s degree or above	17	Senior nurse	Outpatient Service
N16	35	Female	Bachelor’s degree or above	13	Senior nurse	Emergency Department
N17	34	Female	Bachelor’s degree or above	12	Senior nurse	Surgery
N18	24	Female	Bachelor’s degree or above	2	Registered nurse	Internal Medicine
N19	36	Female	Bachelor’s degree or above	13	Senior nurse	ICU
N20	37	Female	Bachelor’s degree or above	15	Senior nurse	Internal Medicine

#### 2.5.2 Interview guide

Drawing on a preliminary literature review, earlier pilot work, and expert consultation, the research team developed a semi-structured interview guide (**Table 2**) to explore nurses’ experiences of caring for patients with mental-physical comorbidity, the challenges they encountered, and the factors shaping these experiences.

**Table 2:** Examples of interview questions.

Questions
How do you view patients with mental-physical comorbidity? How do you identify mental-health problems during routine care? What specific nursing measures do you use when caring for these patients? In your opinion, what aspects of mental-health care are currently insufficient? Which steps in the care process are most challenging? What do you think would improve the quality of care for patients with mental-physical comorbidity? What concrete suggestions do you have for enhancing non-psychiatric nurses’ competence in this area?

### 2.6 Recruitment procedure

Following approval from the nursing administration, study posters describing the purpose and eligibility criteria were displayed in non-psychiatric units. Nurses who were interested and met the criteria contacted the research team directly and were enrolled after eligibility screening.

### 2.7 Data collection

Before interviewing began, an experienced qualitative researcher trained the interviewers in interviewing techniques and research ethics. The interviewers had no prior personal or professional relationship with the participants, did not know them before recruitment, and had no supervisory, managerial, or evaluative role in relation to them. Written informed consent was obtained from each participant after the study aims, procedures, potential risks and benefits, and confidentiality arrangements had been explained. Interviews took place in a quiet private room and were audio-recorded with permission. Interviewers also noted relevant non-verbal cues such as facial expression and body language. Interviews lasted 40–60 minutes. Within 24 hours, two researchers independently transcribed each recording verbatim and incorporated relevant field notes. The independently prepared transcripts were compared line by line to identify omissions or transcription errors. Each verified transcript was returned to the corresponding participant to confirm that it accurately reflected the interview content. All 20 participants completed this transcript-checking process.

### 2.8 Data analysis

Two researchers independently analysed the transcripts using Colaizzi’s seven-step phenomenological method, with NVivo 12.0 used for data organisation (26). They first read each transcript repeatedly to become familiar with participants’ accounts. Significant statements related to the phenomenon were identified, meanings were formulated, and similar meanings were grouped into thematic clusters. The two researchers then compared their analyses, discussed discrepancies, and agreed on the final thematic structure; a third qualitative researcher was consulted when agreement could not be reached. The themes were integrated into an exhaustive description and then condensed into the fundamental structure of the phenomenon. Five participants from different clinical departments were purposively invited to review the preliminary themes. They were asked whether the interpretation reflected their experiences and whether important content had been omitted or misinterpreted. None identified a substantive discrepancy.

### 2.9 Data management

Audio recordings and transcripts were managed under the study’s confidentiality procedures. Audio files were deleted after verbatim transcription and checking. Electronic files were stored in an encrypted folder accessible only to the core research team. Any paper materials were scanned into secure digital files and then shredded. Access to study data was restricted to authorised members of the research team.

### 2.10 Rigour

Rigour was addressed through credibility, confirmability, dependability, and transferability (27). For credibility, all 20 participants checked their transcripts, and five participants from different clinical departments reviewed the preliminary themes; none identified substantive discrepancies. Two researchers analysed the data independently and discussed differences, consulting a third qualitative researcher when needed. During interviewing and analysis, the researchers kept and discussed reflexive notes on assumptions arising from their professional experience and revisited these notes during theme development. Analytic decisions were documented in an audit trail. Consistent procedures for recruitment, interviewing, transcription, and analysis supported dependability. Reporting participant characteristics and clinical departments allows readers to judge the transferability of the findings to other non-psychiatric settings.

### 2.11 Ethical considerations

Ethical approval was obtained from the Medical Ethics Committee of the Affiliated Mental Health Centre & Hangzhou Seventh People’s Hospital, Zhejiang University School of Medicine (Approval No. 2025-033). Before each interview, participants received information about the study purpose, procedures, confidentiality, potential risks and benefits, and the voluntary nature of participation, and provided written informed consent. Participants were identified by codes N1–N20 in transcripts and reports.

## 3 Results

Two domains were identified: (1) experiences and challenges in caring for patients with mental-physical comorbidity and (2) factors shaping these experiences. The first domain comprised seven themes: difficulty recognising psychiatric symptoms; difficulty balancing physical and mental care; safety concerns; limited effectiveness of available interventions; communication barriers; reliance on experiential knowledge; and night-shift challenges. The second comprised six themes: stigma and concealment of mental illness; challenges in engaging patients in care; lack of standardised guidance and mental health knowledge and skills; limited collaboration between psychiatric and non-psychiatric services; limited communication skills; and workload and time pressure.

### 3.1 Experiences and challenges in caring for patients with mental-physical comorbidity

#### 3.1.1 Theme 1: difficulty recognising psychiatric symptoms

Participants described difficulty recognising psychiatric symptoms when signs were subtle or patients were clinically stable. Identification often depended on patient disclosure or psychiatric consultation, making concealed or early symptoms harder to detect.

N8: “I first worked in the emergency resuscitation room and met many psychiatric patients. When they were in an episode, I could recognise their mental illness, but under ordinary circumstances it was hard. I’m not a psychiatric specialist, so I can’t define the exact type of illness.”

N2: “If a tumour patient says they feel unwell, I first suspect tumour compression. Only when test results are normal and discomfort persists do I consider a mental-health problem.”

N10: “A manic or bipolar patient looks normal when not symptomatic; I wouldn’t notice unless they told me. I might identify severe mood instability on admission, but I can’t always spot early anxiety or depression unless psychiatry consults are ordered.”

N15: “The scariest cases are the covert ones. In everyday conversation they seem just slightly indifferent and very compliant. Only after self-harm occurs do we realise they had other plans. That’s extremely hard to detect.”

#### 3.1.2 Theme 2: difficulty balancing physical and mental care

Participants described tension between addressing physical needs and avoiding procedures they feared might intensify distress or psychiatric symptoms. At the same time, untreated physical discomfort could worsen anxiety or behavioural disturbance.

N14: “We are strangers to them; some procedures become extra stimuli. We worry that extra stimulation might worsen their condition.”

N11: “Severely ill patients may attempt suicide. They feel bad but we find no cause, leading to anxiety that can worsen both mental illness and physical disease.”

N19: “Take myocardial infarction or hypertension. If mental illness is present, they repeatedly check blood pressure, and anxiety itself elevates BP, further burdening the vessels.”

#### 3.1.3 Theme 3: safety concerns

Participants described concerns about self-harm, aggression, falls, and unplanned removal of devices. Close-contact care also left some nurses feeling vulnerable to aggression.

N17: “Suicide or self-injury is the greatest risk. Their mood is unstable, plus fall or bed-fall risk. Safety management requires constant vigilance.”

N14: “Some patients act out, harming themselves or relatives. Communication itself can trigger over-reaction; self-harm or aggression is a daily concern.”

N19: “They can’t control themselves during episodes, risking falls and catheter removal. Near-bed nursing exposes us to aggression.”

#### 3.1.4 Theme 4: limited effectiveness of available interventions

Participants described psychiatric consultation and medication as the main responses available during acute episodes, but said these measures were not always sufficient and that they had few alternatives.

N1: “Some patients hit doctors and nurses. We seek psychiatry consults; sedatives help, but once they wear off, agitation returns.”

N2: “Sedatives face tolerance issues; many patients don’t respond quickly, and not everyone benefits.”

N11: “Psych consults mostly prescribe drugs. If medication fails, we have no further strategy. Our ward focuses on anti-tumour care—we lack special measures for mental illness.”

#### 3.1.5 Theme 5: communication barriers

Communication was described as difficult when patients were highly distressed, cognitively impaired, sedated, or reluctant to discuss mental health problems.

N12: “With psychiatric patients, usual techniques fail. Some ignore everything. Many patients and families are reticent about mental illness, so communication is lacking.”

N3: “Some cannot even understand Mandarin, let alone converse when agitated or aggressive.”

N4: “Medications slow their responses; they don’t register what we say.”

N8: “They speak incoherently or mutter to themselves, mixing reality and hallucination. Depressed patients hide, cry quietly, and respond minimally.”

#### 3.1.6 Theme 6: reliance on experiential knowledge

Participants said care often depended on individual experience because formal guidance was limited. More experienced nurses described being more alert to potential risks, whereas less experienced staff tended to focus on routine physical care.

N1: “We depend on experience. Skilled nurses notice dangers and report to doctors; less experienced staff stick to vitals and routine tasks.”

N13: “These cases are rare; handling relies entirely on experience and responsibility. Capable nurses detect more issues; others don’t.”

N3: “I’m older, trained, and sensitive. Younger nurses struggle.”

#### 3.1.7 Theme 7: night-shift challenges

Participants viewed night shifts as particularly difficult because behavioural or psychiatric symptoms could become more apparent while staffing was reduced. Some reported responsibility for 50–70 patients, which limited close observation.

N7: “Dementia symptoms intensify at night. I cover 50–70 patients alone; it’s frightening and stressful.”

N14: “One patient fell from the second floor after hearing voices; another overdosed at night. Such risks are real.”

N4: “Night staffing is one nurse per 50-plus patients; we must patrol hourly. When I’m away, high-risk patients roam.”

N10: “They may attempt self-harm, but I can’t watch one patient 24/7 when I have 60 others.”

### 3.2 Factors shaping nurses’ experiences

#### 3.2.1 Theme 1: Stigma and concealment of mental illness

Participants described stigma as a reason some patients and families withheld psychiatric histories or avoided discussing mental health problems. This made it harder for nurses to obtain complete information and assess care needs.

N9: “Some people hide their psychiatric history; they also do not want to talk much about mental-health problems. No one is willing to admit that they have a mental problem. Almost everyone chooses to conceal it. Even someone who already has an anxiety disorder may not realise that anxiety is a mental problem.”

N6: “Many patients are not symptomatic all the time, so even if they have a mental illness, they can appear normal—especially those who have not completely lost their ability to work. They still need to fit into the workplace and fear discrimination, so they keep the mental illness very private; they see it as something to avoid at all costs.”

#### 3.2.2 Theme 2: challenges in engaging patients in care

Participants described situations in which psychiatric symptoms, distress, or impaired comprehension made it difficult to engage patients in treatment and nursing care, particularly during acute episodes.

N16: “When we care for delirious patients, some are very unco-operative and pull out lines such as their catheters, cannulas, and even punch or kick nurses.”

N20: “When caring for them, the first impression is that their co-operation is much worse and their comprehension much poorer. The symptoms they describe or changes in their physical condition can further worsen their anxiety, so their co-operation is greatly reduced.”

#### 3.2.3 Theme 3: lack of standardised guidance and mental health knowledge and skills

Participants reported few clear protocols to guide care. Even when screening tools were available, they were uncertain about what actions should follow a positive finding. They also described gaps in mental health assessment, communication, psychotropic medication knowledge, and adverse-effect monitoring.

N5: “For us non-psychiatric nurses, what we lack is a standardised nursing guideline. For example: What symptoms does this disease have? What solutions are available? Just like internal-medicine or surgical diseases—what symptoms, how to treat, how to nurse. We need something plain, simple, that can truly solve problems and be used in clinical practice. Right now we rely entirely on experience and our own thinking, but it always feels like something is missing. We need a comprehensive plan to manage these comorbidity patients well.”

N19: “My theoretical knowledge of mental health isn’t particularly strong; I can only combine it with my own experience. We also lack targeted psychological guidance. Senior psychological counsellors have a whole series of courses; we nurses lack systematic training. We can’t be keenly aware of problems when we meet such patients in clinical practice without professional knowledge.”

N4: “I think mental and physical illnesses interact and complement each other. For example, in our department, insomnia is related to sudden deafness or tinnitus. The ear keeps ringing at night, and the patient can’t sleep, which is painful. But in such situations we feel powerless because we lack knowledge and skills to handle the mental illness.”

N8: “For example, knowledge of depression, anxiety, and schizophrenia; and oral medications—we need to know what the drug does and possible side-effects. Right now we don’t know these things. If a delirious patient is given a sedative, what side-effects might occur? Not everyone knows.”

#### 3.2.4 Theme 4: limited collaboration between psychiatric and non-psychiatric services

Participants described collaboration with psychiatric services as limited and sometimes difficult to translate into ward-based care because psychiatric and non-psychiatric services operated with different priorities and workflows.

N14: “Psychiatry is a very specialised and independent department. For example, I’m an emergency or ICU nurse; when I rotate to endocrinology, haematology, cardiology, basic nursing work is continuous. But in psychiatry, the nursing work cannot co-operate effectively. Even if we ask for a psychiatric consult, the advice is hard to implement and has very little effect.”

N1: “Some patients strongly resist treatment and won’t let me touch them; if I get close, they want to hit me. Even after a psychiatric consult, their solutions don’t integrate well with our clinical work and don’t achieve the expected effect—so we can’t form effective co-operation.”

N11: “Whether it’s our doctors and nurses or psychiatric staff, everyone’s goal is to manage the patient well, but because of the special circumstances of these patients and various limitations, we can’t achieve the desired effect.”

#### 3.2.5 Theme 5: limited communication skills

Participants reported difficulty explaining mental health problems to patients and families and managing conversations when emotions were intense. They described a need for more specific communication and de-escalation skills.

N8: “During the explanation process, I felt I didn’t use good verbal communication skills to let him realise he is in a disease state.”

N18: “Communication skills are very important. They may not like to talk or may talk rapidly and emotionally; you need good skills. Emotional-regulation ability is also key: You must stay calm and rational immediately; you can’t argue with an emotional patient.”

#### 3.2.6 Theme 6: workload and time pressure

Workload and time pressure left little room for mental health assessment, supportive communication, or ongoing observation in some units. Nurses said physical care tasks often took priority during busy shifts.

N12: “Surgical work is fast-paced and busy, so we don’t have much time to pay attention. We just finish treatments quickly and meet the workload; there’s no excess time to focus on them. It’s the same in other departments—little time with patients, which directly affects nursing quality.”

N20: “Documentation takes a lot of time; real bedside communication is little. We work mechanically: at this time I make rounds; at that time I do cord-care for babies. We don’t truly spend time with patients.”

N5: “We’re busy. Spending a lot of time counselling patients isn’t possible. Our ward has 66 beds; each nurse cares for more than ten patients plus admissions and discharges. So I can provide physical care, but for mental-symptom care, time isn’t enough. Completing physical-care tasks during shifts is already a lot of pressure.”

## 4 Discussion

Non-psychiatric nurses described clinical uncertainty and organisational constraints that complicated care for patients with mental-physical comorbidity. Difficulties were most evident when psychiatric symptoms were subtle, mental and physical priorities competed, safety concerns required close observation, or night-shift staffing limited time with patients. Participants linked these experiences to gaps in mental health knowledge and communication skills, limited guidance, difficulties obtaining workable psychiatric support, and workload pressure.

Knowledge gaps were evident across symptom recognition, communication, risk management, and medication-related care, consistent with research linking mental health literacy to recognition and management of mental disorders (28). Participants had particular difficulty recognising psychiatric symptoms when presentations were subtle or stable (22) and often relied on prior experience and obvious behavioural changes. Stigma further limited access to clinical information because some patients and families were reluctant to disclose psychiatric histories (29). Similar research suggests that reluctance to accept or disclose a psychiatric label can delay help-seeking (30). In this setting, recognition depends on both nurses’ preparation and the information available during routine assessment. Training should therefore include common psychiatric presentations, warning signs, and structured assessment approaches relevant to general hospital care.

Participants also reported limited confidence in communication, suicide prevention, and the management of aggressive behaviour, consistent with previous research on preparedness among non-psychiatric nurses (31). The lack of clear protocols intensified this uncertainty because nurses often relied on personal experience to decide what to do. Broader work has also identified gaps in standards for assessing mental health care quality (32). In general hospital settings, protocols need to extend beyond screening by specifying the response to a positive finding, thresholds for psychiatric consultation, and immediate safety measures. This would give training and competency assessment a clearer clinical basis.

Previous studies have reported that non-psychiatric nurses may treat mental and physical problems as separate areas of care (33, 34). Participants in this study generally recognised that the two could influence one another, but this recognition did not remove practical uncertainty. They worried that routine procedures might intensify distress or psychiatric symptoms, while physical discomfort could aggravate anxiety or behavioural disturbance. Their accounts suggest awareness of the need for integrated care without clear strategies for balancing competing priorities. Difficulty engaging patients during acute psychiatric symptoms added to this challenge (35).

Earlier studies have focused mainly on nurses’ knowledge, competence, and attitudes (31, 33, 34). Participants in this study also described organisational barriers at the interface between psychiatric and non-psychiatric services (36). Multidisciplinary collaboration can be difficult to establish and sustain (37), and much of the literature concerns collaboration within somatic specialties or within psychiatric care (38–41). In general hospital units, psychiatric recommendations may be difficult to implement when they do not fit local workflows, staffing patterns, or the immediate demands of physical care. Consultation pathways, shared care protocols, and direct communication between psychiatric and non-psychiatric teams may therefore be more useful than *ad hoc* referral alone.

Time pressure was another recurring concern. Similar studies have shown that limited time and staffing constrain observation and interaction with patients who have mental health needs (42, 43). Participants described this problem as especially pronounced on night shifts, when one nurse could be responsible for a large number of patients. Staffing models that better reflect patient acuity and mental health risk may give nurses more capacity for observation, communication, and timely response, although such approaches require evaluation in different service settings.

Training for non-psychiatric nurses should focus on recognising common psychiatric presentations, responding to self-harm or aggression risk, communicating with distressed patients, and monitoring psychotropic medication effects. Previous work suggests that targeted education can improve nurses’ knowledge, attitudes, and confidence (19, 44). Case-based and ward-based formats may be more readily applicable than generic mental health content. Psychiatric support also needs to be accessible in routine practice. Options include clinical rotations, on-site consultation, case discussion, and shared communication channels (37). Local protocols could set out screening, escalation, consultation, and safety procedures, with risk checklists adapted from existing work (45). Staffing and workload should be considered alongside training; adjusting night-shift staffing or nurse-patient allocation in response to patient demand may create more time for observation and communication (46).

## 5 Limitations

Several limitations should be considered. Participants were recruited from one tertiary hospital in Hangzhou, so the findings may not transfer to other institutions, regions, or healthcare systems. The sample was predominantly female, with 19 women and one man, which may limit the range of gender-related perspectives represented. Recruitment relied on voluntary responses to study posters. Nurses with particularly strong experiences or views about caring for patients with mental-physical comorbidity may have been more likely to participate, creating potential self-selection bias and narrowing the range of perspectives represented. Participants worked in different clinical departments, and differences in patient populations and workflows may have shaped their accounts. Future work could examine particular departments in greater depth and include nurses from multiple institutions and regions. Finally, interviews were conducted in Chinese. Translating participant quotations into English may have introduced minor differences in meaning.

## 6 Conclusion

This study described non-psychiatric nurses’ experiences of caring for patients with mental-physical comorbidity and the factors shaping those experiences. Participants reported difficulty recognising psychiatric symptoms, balancing mental and physical priorities, managing safety, communicating with patients, and allocating time for mental health care. These challenges were linked to limited training and guidance, weak integration with psychiatric services, and workload pressure. Multicentre research is needed to examine how these experiences vary across settings. The findings identify practical areas for nursing education, clinical guidance, consultation pathways, and workforce support.

## Data Availability

The qualitative data generated and analyzed during the current study are not publicly available because they contain potentially identifiable interview information. De-identified data may be available from the corresponding authors upon reasonable request and subject to applicable ethical and institutional requirements.
